# Microevolution of *Puumala hantavirus* during a Complete Population Cycle of Its Host, the Bank Vole (*Myodes glareolus*)

**DOI:** 10.1371/journal.pone.0064447

**Published:** 2013-05-22

**Authors:** Maria Razzauti, Angelina Plyusnina, Heikki Henttonen, Alexander Plyusnin

**Affiliations:** 1 Department of Virology, Haartman Institute, University of Helsinki, Helsinki, Finland; 2 Finnish Forest Research Institute, Vantaa, Finland; Duke-NUS Gradute Medical School, Singapore

## Abstract

Microevolution of *Puumala hantavirus* (PUUV) was studied throughout a population cycle of its host, the bank vole (*Myodes glareolus*). We monitored PUUV variants circulating in the host population in Central Finland over a five-year period that included two peak-phases and two population declines. Of 1369 bank voles examined, 360 (26.3%) were found infected with PUUV. Partial sequences of each of the three genome segments were recovered (approx. 12% of PUUV genome) from 356 bank voles. Analyses of these sequences disclosed the following features of PUUV evolution: 1) nucleotide substitutions are mostly silent and deduced amino acid changes are mainly conservative, suggesting stabilizing selection at the protein level; 2) the three genome segments accumulate mutations at a different rate; 3) some of the circulating PUUV variants are frequently observed while others are transient; 4) frequently occurring PUUV variants are composed of the most abundant segment genotypes (*copious*) and new transient variants are continually generated; 5) reassortment of PUUV genome segments occurs regularly and follows a specific pattern of segments association; 6) prevalence of reassortant variants oscillates with season and is higher in the autumn than in the spring; and 7) reassortants are transient, i.e., they are not competitively superior to their parental variants. Collectively, these observations support a quasi-neutral mode of PUUV microevolution with a steady generation of transient variants, including reassortants, and preservation of a few preferred genotypes.

## Introduction

Hantaviruses constitute a distinct genus within the *Bunyaviridae* [1]. This family is represented by enveloped negative-stranded RNA viruses that possess a tri-segmented genome: the small (S) segment encodes the nucleocapsid (N) protein and, in some hantaviruses, also a non-structural protein (NSs); the medium (M) segment encodes two surface glycoproteins (Gn and Gc); and the large (L) segment encodes the L protein, the viral RNA-dependent RNA polymerase [2].

*Puumala virus* (PUUV) [3] is the main rodent-borne pathogen in Europe, where it causes *nephropathia epidemica* (NE), a relatively mild form of haemorrhagic fever with renal syndrome [4]. The host of PUUV is the bank vole (*Myodes glareolus*), a woodland rodent that occurs from the British Isles through continental Europe and Russia to the Lake Baikal [5]. The occurrence of NE depends strongly on the population dynamics of the bank vole; population peaks are mirrored by NE outbreaks with a short time-lag [6]–[8].

Bank voles are chronically infected by PUUV [9]–[10]. Viral secretion occurs during half a year after infection [9], [11] and the virus transmission is horizontal, mainly via breathing of aerosolized excreta generated by infected rodents [12]–[14]. Such indirect mode of transmission and virus survival outside the host promotes viral persistence in the bank vole population [15]–[16]. Maternal antibodies transferred by infected females to their progeny protect them up to 80 days, and this may shape the dynamics of virus transmission during breeding season and shortly thereafter [17]–[18]. Similar to other RNA viruses, hantaviruses exhibit a high short-term substitution rate. Genetic diversity in hantaviruses is generated by genetic drift (i.e., a gradual accumulation of point mutations throughout the genome coupled with small deletions and insertions within the non-coding regions of the RNA segments [19]) and reassortment of genome segments [19]–[22]. Evidence for homologous recombination has also been reported [23]–[24], but this seems to be a rare event [25]. While the vast majority of point mutations observed are silent, reflecting stabilizing selection at the aa level [26], there is some evidence for directional selection [27]. Genetic heterogeneity is comparable among hantaviruses species found in different rodent subfamilies. Nucleotide (nt) diversity among hantavirus species is variable and characterized by the number of strains recognized. Inter-lineage diversity of PUUV may be as high as 38% for the S segment, and sequence heterogeneity is unevenly distributed along the genome [28]. Despite the growing database of genome sequences and the increasing number of studies on genetic variability of hantaviruses, none to date have considered the nature of viral genetic diversity on a scale of a host population and sampled during several-years period. However, such studies of virus microevolution are essential for a better understanding of the mechanisms shaping hantavirus evolution. Previously [19], we examined PUUV genetic diversity in a bank vole population. This study included 31 distinct viral strains circulating during 2005 in Central Finland. A substantial genetic diversity was observed between circulating PUUV strains. Moreover, phylogenetic analysis of the S, M and L genome sequences clustered the variation into two distinct genogroups. As such, recognition of reassortant strains was straightforward and 20% of the circulating PUUV strains were found to be carrying inter-genogroup reassortant genomes. This analysis raised several questions concerning temporal fluctuations of the diversity of PUUV strains, how it is influenced by the host population density, and the fate of individual PUUV strains. Here, we expand our earlier study and report the microevolution of PUUV in its host population during a five-year period, covering both peak and decline phases of the bank vole population cycle.

## Materials and Methods

### Ethics statement

According to the Finnish Act on the Use of Animals for Experimental Purposes (62/2006) and a further decision by the Finnish Animal Experiment Board (May 16th, 2007), the techniques employed to capture rodents, i.e., life- and snap-trapping are not considered an animal experiment and therefore requires no animal ethics license from the Finnish Animal Experiment Board. The species captured for this study, *Myodes glareolus*, neither is protected nor included in the Red List of Finnish Species. Animal trapping took place on private and Finnish national forest by permit (1013/204/2002). Landowners were consulted and the trapping was allowed before the study was conducted.

### Sampling of rodents

Rodents were trapped at Konnevesi, Central Finland (62°34′N, 26°24′E) twice a year (May and October) from 2005 till 2009. During the early 2000s, bank voles were strongly cyclical in Central Finland [7]; 2005 was a cyclic peak year, 2006 decline and crash, 2007 a strong increase phase, 2008 a high peak, and 2009 another decline. Trapping was done at 38 sites within 120 km^2^ of typical taiga forest, mainly dominated by Norway spruce (*Picea abies*), Scots pine (*Pinus sylvestris*), downy birch (*Betula pubescens*) and silver birch (*Betula pendula*). Of 38 trapping sites, 14 were sampled from May 2005 to October 2009 using grids of 3×3 Ugglan Special live traps (Grahnab, Hillerstorp, Sweden) set 15 m apart. Traps were set for three nights and checked twice per day to minimize animal stress. From May 2007 onwards, 24 additional trapping sites were added to increase the study material. These consisted of 4 transects of 15 standard snap-traps at 15 m intervals over two nights. The trapping sites were situated 500 to 1000 m apart from each other. Bank voles captured alive were anesthetized with isofluarene (Forene, Abbott, UK), bled from the retro-orbital sinus and sacrificed by cervical dislocation and immediately frozen. Similarly, snap-trapped bank voles were flash-frozen on dry ice until necropsy. The dissection of rodents was performed in a class II laminar flow hood in a biosafety level 3, animal annotation (weight, sex, maturity, age) was completed and tissue samples were individually deep-frozen until further analyses.

### Screening of samples

All rodents were first screened for the presence of PUUV N-antigen (Ag) using immunoblotting. Briefly, lung tissue samples (approx. 20 mg) in 500 µl of Laemmli buffer were incubated at room temperature overnight, then homogenized by sonication and heated to 80–100°C for 5 minutes. Aliquots of 25 µl were separated by electrophoresis in 10% sodium dodecyl sulphate polyacrylamide gels and proteins were subsequently transferred onto nitrocellulose membranes. To verify the blotting efficacy, membranes were stained with 1× Ponceau S staining solution for 1 minute and destained in distilled water until the background was clear. Membranes were blocked with 1% bovine serum albumin solution overnight at +4°C. Membranes were incubated with rabbit polyclonal antiserum made against recombinant N-Ag for two hours at room temperature, washed with 0.1% Ten-Tween20 solution, and incubated for one hour with Odyssey IRDye 800CW goat anti-rabbit secondary Ab (LI-COR), diluted at 1∶10000 in PBS. Odyssey Infrared Imaging system was used to detect blotted proteins.

### RT-PCR and sequencing of the PUUV genome

Viral RNA was extracted from lung tissue samples of the N-Ag-positive bank voles using the TRIsure reagent (Bioline, UK) according to the manufacturer's instructions. Reverse transcription was performed with RevertAid™ H Minus M-MuLV Reverse Transcriptase (Fermentas, Lithuania) and AmpliTaq® DNA polymerase (Applied Biosystems, Foster City, CA, USA) was used to amplify viral cDNA. PUUV S- (nt 631–1085), M- (nt 2162–2613) and L-segment (nt 505–1040) sequences were amplified as described earlier [19]. PCR amplicons were purified with ExoSAP-IT™ PCR clean-up reagent (USB Corporation, Miles Road, Cleveland, USA). Automated sequencing was performed using the ABI PRISM™ Dye Terminator sequencing kit (Perkin Elmer/ABI). Newly recovered S-, M- and L-segment sequences (455, 452 and 536 nt in length respectively) were deposited in GenBank under accession numbers JQ319161–JQ319319.

### Genetic variation and phylogenetic analysis

Nucleotide sequence alignments were generated with BioEdit v7.0.9 [29]. Genetic distances were estimated within and between viral populations with DnaSP [30]. PHYLIP program package [31] was employed to estimate phylogeny of the PUUV sequences. Genetic distances were calculated with the F84 substitution model (Dnadist) and bootstrap supports were obtained generating 1000 replicates of the datasets (Seqboot) and for the reconstruction of PUUV phylogenies the Neighbour-joining (NJ) algorithm (Neighbor) was used. Sequence alignments were submitted to phylogenetic Network 4.600 software (Fluxus-Engineering) to generate genetic and evolutionary relationships among the segment genotypes using Median-Joining algorithm [32]. An examination of recombination among complete sequences was performed using the recombination detection program (RDP) [33] and SimPlot v3.5.1 [34].

## Results

### PUUV prevalence and genetic diversity in the bank vole population at Konnevesi

The incidence of PUUV infection and genetic variation of the virus were monitored through a complete bank vole population cycle at Konnevesi from May 2005 until October 2009. The study period covered two peak-phases in 2005 and 2008, and two population declines in 2006 and 2009 (presumably followed by virus bottlenecks). A total of 1369 bank voles were captured, of which 360 (26.3%) were PUUV-N-Ag-positive. PUUV prevalence at Konnevesi was higher in spring (a mean of 39%) when most voles in the population have over-wintered rather than in autumn (20.7%) when younger animals dominate (Table 1).

**Table 1:** Number of bank voles captured during the study, PUUV prevalence and proportions of A and B genogroups and reassortants at Konnevesi.

	May	Oct.	May + Oct.	May	Oct.	May	Oct.	May	Oct.	Total	
	2005		2006	2007		2008		2009			
**no. of trapped bank voles**	47	100	8	54	132	237	625	78	88	1369	
**no. of PUUV+**	22	22	0	7	15	106	155	28	5	360	
**(%)**	(46.8)	(22)		(13)	(11.4)	(44.7)	(24.8)	(35.9)	(5.7)	(26.3)	
**no. of variants of genogroup A**	5	4	0	2	5	40	51	13	5	125	
**(%)**	(27.8)	(18.2)		(28.6)	(33.3)	(37.7)	(32.9)	(46.4)	(100)	(35.1)	
**no. of variants of genogroup B**	10	13	0	4	7	53	67	9	0	163	
**(%)**	(55.6)	(59.1)		(57.1)	(46.7)	(50)	(43.2)	(32.1)		(45.8)	
**no. of Reassortant variants**	3	5	0	1	3	13	37	6	0	68	
**( %)**	(16.7)	(22.7)		(14.3)	(20)	(12.3)	(23.9)	(21.4)		(19.1)	

**Figure 1:**
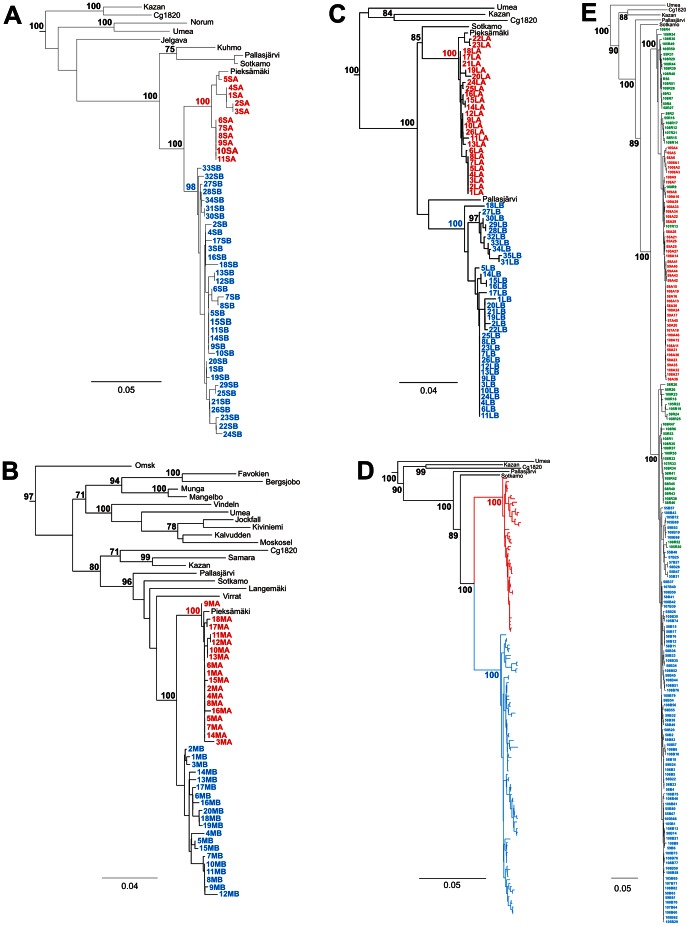
Phylogenetic trees. Phylogenetic trees (Neighbor-Joining) calculated for the S, M and L segment sequences of PUUV, and contings of the three segment sequences: (A) partial S (nt 631–1085); (B) partial M (nt 2162–2613); (C) partial L (nt 505–1040); (D) a contig of the three genome segments omitting reassortant variants, and (E) a contig of the three genome segments including reassortant variants. A maximum clade credibility tree with an arbitrary root is shown with mean branch lengths (substitutions per site), and non-parametric bootstrap percentages are shown for each node. DOBV, ANDV, SNV and TULV were used as outgroups and omitted for the graphical representation.

Partial genome sequences were recovered from 356 PUUV N-Ag-positive bank voles: 455 nt for the S segment, 452 nt for the M segment and 536 nt for the L segment. Direct sequencing of PCR-products was employed; this way the master sequences were recovered and the “noise” of quasispecies ignored. Taken together, partial S-, M- and L-sequences (a total of 1443 nt) represented 12% of the PUUV genome. Of 356 PUUV genomes, M-segment sequences were recovered for 353 genomes (interestingly, all the remaining were reassortant). Pairwise sequence analysis revealed 182 nt differences in the segments analyzed: 53 mutations in the S, 50 in the M, and 79 in the L. The majority of mutations were silent and occasionally non-synonymous substitutions were encountered (Table 2). Numerous genetic markers were found along the studied segments: 10 for the S (T699C, T750C, T789A, A810G, G843A, T876C, G936A, T945C, C1014T, T1044C), 7 for the M (C2180T, C2207T, A2216G, G2471A, A2483G, G2543A, C2567T) and 29 for the L (G514T, G523A, T532C, C538T, G656A, C593T, G601A, G679A, C682T, A742G, A754G, G769A, C829T, A832G, T838C, G904A, T910C, G916A, A922G, C928T, A964T, C967T, A982T/G, A988T/C, A1003G, A1006G, C1009T, T1025C, C1034T) (Table 2). Such genetic markers allowed the recognition and discrimination of two genogroups (i.e., genetically related strains within a virus lineage) referred as “A” and “B” and represented in the figures in red and blue colors respectively. These groups were also inferred from phylogenetic analyses of sequence data (Fig. 1).

**Table 2:** Genetic diversity of PUUV variants at Konnevesi.

Genome segment		S	M	L
**Length**	nt	455	452	536
	aa	151	150	178
**no. of nt substitutions**	Groups **A** and **B**	53	50	79
	Within group **A**	17	16	24
	Within group **B**	33	32	38
**Genetic diversity (%)**	Groups **A** and **B**	6.2	4.8	10.1
	Within group **A**	2.5	1.6	1.5
	Within group **B**	2.5	2.7	3.2
**no. of aa substitutions**	Between groups **A** and **B**	-	-	1 (**A** I83V **B**)
	Within group **A**	3 (K41R, M75I, D79E)	3 (S11N, E43K, C109G)	3 (I43V, N93I, N168K)
	Within group **B**	9 (F31I, V33E, R39K, P57S, Q78P, D79N, A87V, A97S, Q144P)	4 (P71L, S92P, S101A, I110V)	8 (T7S, D46E, R67C, N86D, R116K, T121I, S141G, V165I)
**no. of genotypes**	Total	45	38	61
	Within group **A**	11	18	26
	Within groups **B**	34	20	35
**no. of genogroup discriminative markers**	Between groups **A** and **B**	10	7	29

Genetic diversity of PUUV segments within genogroups was similar for all segments (1.5–3.2%). Inter-genogroup diversity varied from 4.8 to 10.1%; surprisingly, the highest values were attributed to the L segment. Furthermore, a non-synonymous substitution was found in the L protein that could be used as a molecular marker for genogrouping; in the genogroup A, the aa residue at position 83 was isoleucine while in the genogroup B this residue was valine (Table 2).

### Genotyping of Konnevesi PUUV strains

**Figure 2:**
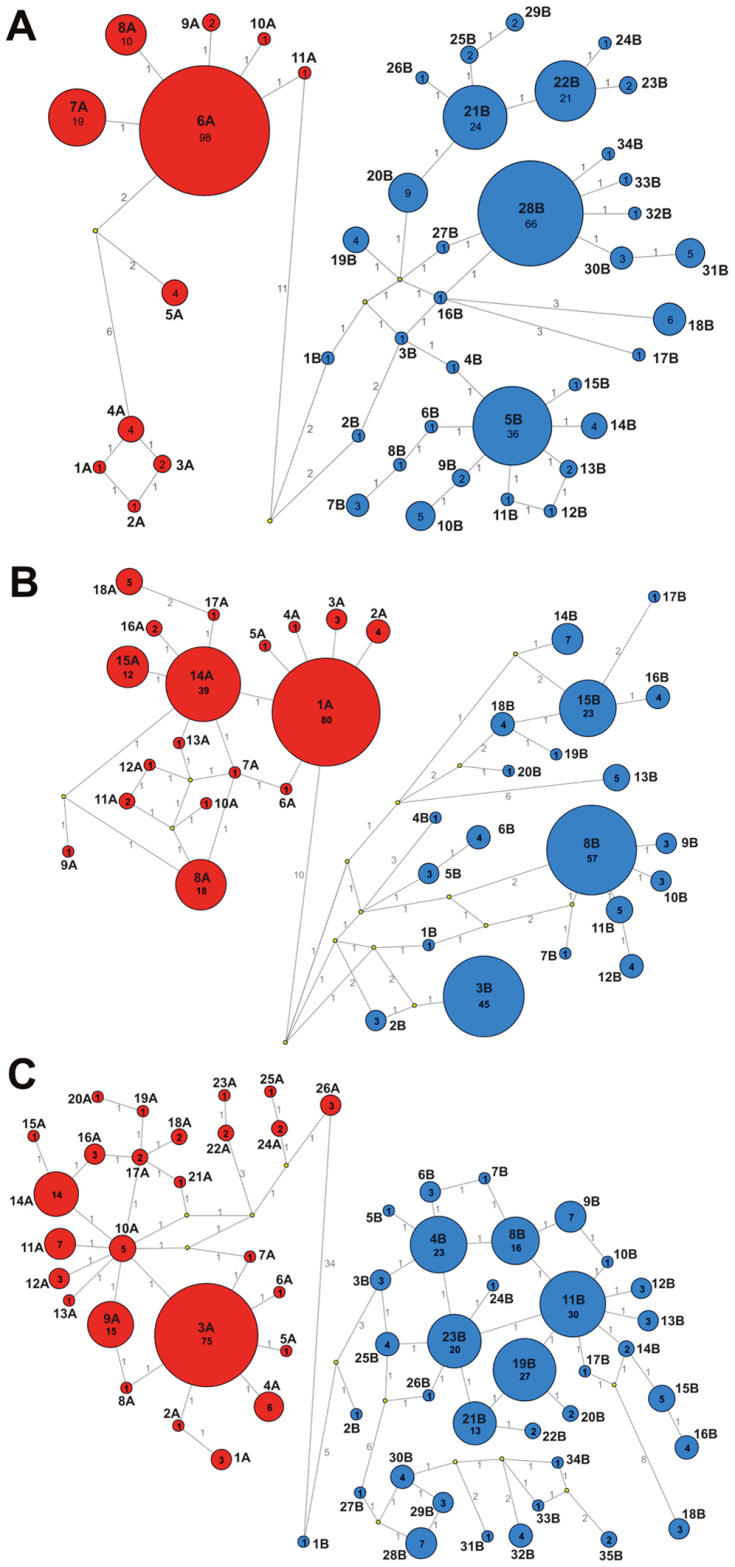
Genetic relationships between (A) S-segment, (B) M-segment and (C) L-segment genotypes. Relationships were constructed using Network analysis and the MJ algorithm. Genotypes of group A are represented by red circles and genotypes of group B by blue circles. The size of the circle is proportional to the number of representatives of each genotype; and numbers are shown inside. Yellow circles are median vectors suggested by Network. The numbers on the link-lines show the number of mutations between genotypes; the length of link-lines is not proportional to the number of mutations.

Genetic diversity of circulating PUUV strains was evaluated, and the number of circulating genotypes (i.e., genetically unique representatives of each of the virus segments) estimated. A total of 45 S-segment genotypes, 38 M-segment genotypes and 61 L-segment genotypes were recognized. Genogroup A included 11 S-, 18 M-, and 26 L-segment genotypes referred respectively as S1A to S11A, M1A to M18A and L1A to L26A. Corresponding numbers for genogroup B were 34, 20 and 35 and respectively referred as S1B to S34B, M1B to M20A and L1B to L35B (Table 2 and Fig. 1). Variants of genogroup B were generally more abundant and diverse (Table 1 and Fig. 2). The 356 PUUV sequences comprised 184 distinct genetic variants. Of those, 46 belonged to genogroup A (A_S_A_M_A_L_), 82 to genogroup B (B_S_B_M_B_L_), and 56 were reassortants between these groups (Fig. 1E). Interestingly, only 39 of these 184 distinct variants were found repeatedly over the observation period; other variants were registered only once (Table 3). No accumulation of certain variants at the given geographic sites was observed. Neither substantial deviation in diversity between sites nor any bias linked to the terrain topology was registered.

**Table 3:** Genetic variants of PUUV observed in the Konnevesi bank vole population.

	Group A	Group B	Reassortant	Total
**Total no. of observed PUUV genomes**	125	163	68	356
**Total no. of PUUV genetic variants**	46	82	56	184
**no. of variants observed more than once (%)**	14 (30.4)	22 (26.8)	3 (5.4)	39 (21.2)
**no. of transient variants (%)**	32 (69.6)	60 (73.2)	53 (94.6)	145 (78.8)

An independent analysis of each segment of PUUV genomes allowed the distinction of the most abundant genotypes (from here on referred to as “copious”): 6A, 7A, 8A, 5B, 20B, 21B, 22B and 28B for the S segment; 1A, 8A, 14A, 15A, 3B, 8B and 15B for the M segment; and 3A, 9A, 14A, 4B, 8B, 11B, 19B, 21B and 23B for the L segment. Figure 2 shows the frequency of occurrence of the segment genotypes as well as their relationships. Clusters of several genotypes can be seen for the S and M segments of both genogroups. These clusters consist of at least one *copious* genotype and several genotypes that were seldom observed (from here on referred to as “sporadic”). For example, the *copious* S genotype 28B and *sporadic* genotypes 27B, 30B, 31B, 32B, 33B, 34B form such a cluster. Differently, the L segment was represented by more genotypes, probably reflecting a higher nt substitutions rate in this segment. Thus high number of drifted nt resulted in abundant genetically-related variants illustrated in interconnected circles of a smaller size in the graphical representation of the segment genotypes (Fig. 2).

### Dynamics of PUUV variants during a bank vole population cycle

**Figure 3:**
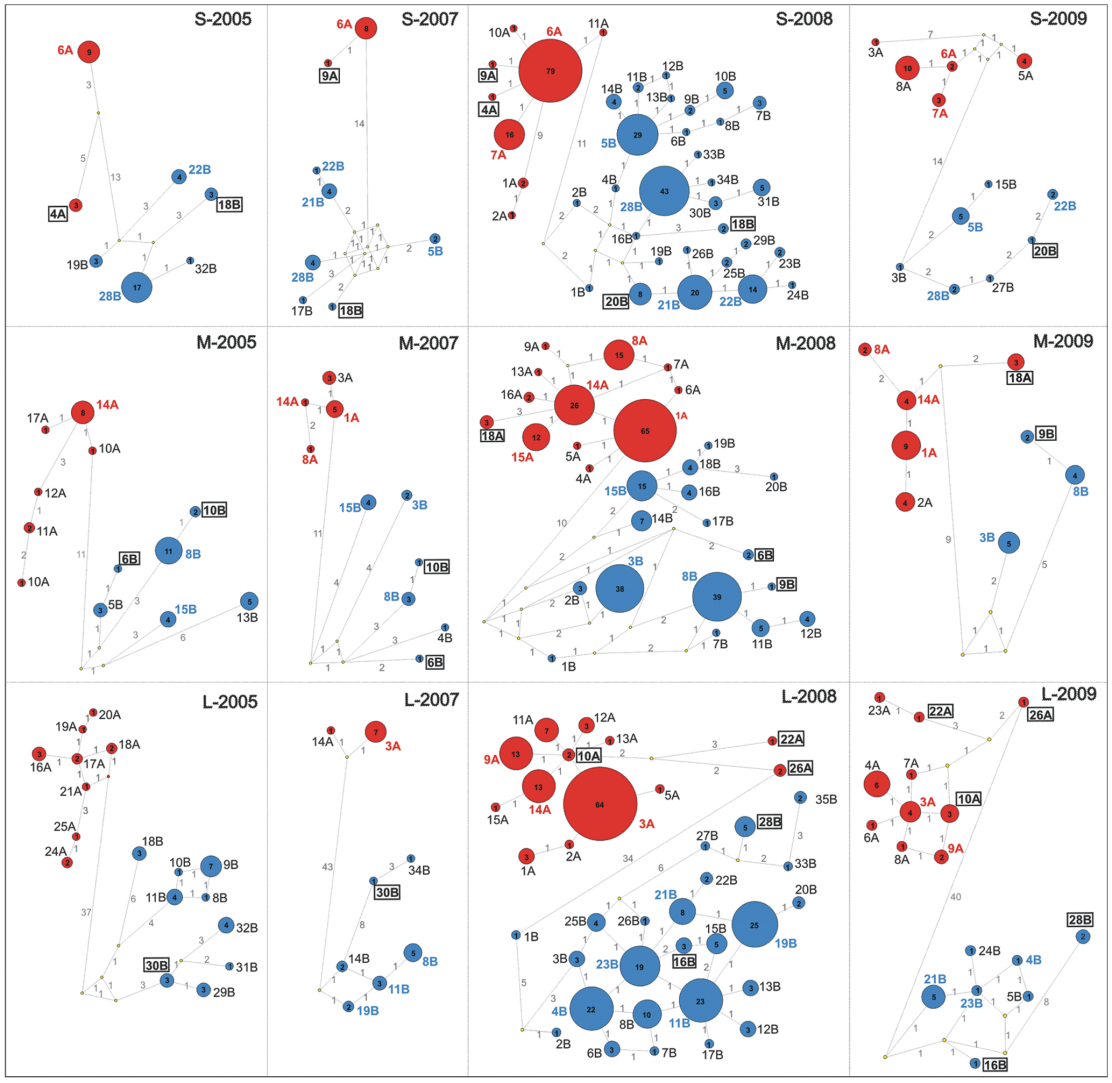
PUUV segment genotypes circulating in the bank vole population at Konnevesi from 2005 to 2009. Relationships between S-, M- and L-segment genotypes was constructed using Network. Genotypes of group A are represented by red circles and genotypes of group B by blue circles. The size of the circles is proportional to the number of representatives of each genotype; and numbers are shown inside. Most abundant circulating genotypes (*copious*) are designated in red and blue, for the A and B genogroups, respectively. *Sporadic* genotypes, detected only once, are shown in black and *sporadic* genotypes observed through several seasons are shown in a square. Yellow circles are median vectors suggested by Network. The numbers on the link-lines show the number of mutations between genotypes; the length of link-lines is not proportional to the number of mutations. Note that the trapping effort was increased from 2007 thus numbers from 2005 are not directly comparable to those of 2007–09.

Relatively large collection of PUUV sequences recovered over a 5-year period gave a unique opportunity to follow individual genetic variants of the virus through phases of high and low population density. Analysis of segment genotypes dynamics revealed that the *copious* genotypes were prevalent throughout the observation window whereas most of *sporadic* genotypes were transient, only a few of them were observed on more than one occasion. New genotypes were continually detected during the study, except in 2006 when no PUUV-infected voles were found (Fig. 3). In other words, the PUUV genes pool was unique at every time-point. Table 4 shows variants that have derived from one or a few nt substitutions, reflecting the contribution of genetic drift to PUUV diversity. Importantly, no evidence for a founder effect (the loss of genetic variation that occurs when a subpopulation of a small number of individuals is established [35]) was observed for the segment genotypes after a decline in the bank vole population (presumably a virus bottleneck). To analyze PUUV strains dynamics, we classified circulating variants into four categories: (i) the most frequently occurring variants, each detected five or more times; (ii) repeatedly observed variants, detected 2–4 times; (iii) transient variants, detected only once; and (iv) reassortant variants, which were analyzed separately due to their peculiar genetic nature (Table 5). The most frequently occurring variants (≥5 times) included only *copious* genotypes for each genome segment. Two of these variants were clearly dominant: *A13* was observed 53 times from May 2007 to October 2009, and *B3* was observed 21 times from October 2007 to October 2008. Repeatedly observed variants (2–4 times) were detected at no more than two study points (Table 4). Of those repeatedly observed variants, 74% of S segments, 67% of M segments and approx. 30% of L segments were of copious genotypes. For the transient variants (1 time), all three segments were represented by *copious* and *sporadic* segment genotypes in approximately equal proportions.

**Table 4:** PUUV variants repeatedly detected in the bank vole population.

no. of representatives	variant	Genotypes			no. of trapping sites	trapping seasons
		S	M	L		
2	***A4***	4	11	24	1	5/05; 10/05
3	***A11***	**6**	**1**	1	2	10/08
53	***A13***	**6**	**1**	**3**	17	5/07; 10/07; 5/08; 10/08; 5/09
2	***A16***	**6**	**1**	10	2	5/08; 10/08
2	***A24***	**6**	**14**	**14**	2	5/08; 10/08
2	***A25***	**6**	**14**	16	2	5/05; 10/05
2	***A27***	**6**	**14**	18	1	10/05
2	***A29***	**6**	**15**	**9**	2	5/08
5	***A30***	**6**	**15**	**14**	4	5/08; 10/08
8	***A34***	**7**	**14**	**9**	2	5/08; 10/08
4	***A36***	**7**	**14**	11	2	5/08; 10/08
2	***A40***	**8**	**1**	**3**	1	5/09
4	***A41***	**8**	**1**	4	1	5/09
2	***A43***	**8**	2	4	1	5/09
2	***B22***	14	**3**	**19**	1	10/08
21	***B3***	**5**	**3**	**19**	7	10/07; 5/08; 10/08
6	***B5***	**5**	**3**	**21**	5	10/08; 5/09
2	***B8***	7	**3**	**21**	1	10/08
2	***B28***	18	6	35	1	5/08
3	***B34***	20	14	15	1	5/08
2	***B35***	20	14	16	2	10/08
5	***B39***	**21**	**15**	**11**	4	10/07; 5/08
2	***B40***	**21**	**15**	14	1	5/07
6	***B42***	**21**	**15**	**23**	2	10/08
3	***B48***	**22**	**15**	29	3	5/05
3	***B59***	**28**	**8**	3	1	10/08
10	***B60***	**28**	**8**	**4**	5	10/08; 5/09
3	***B73***	**28**	11	**4**	2	10/08
4	***B78***	31	12	**4**	2	10/08
2	***B62***	**28**	**8**	6	1	10/08
12	***B64***	**28**	**8**	**8**	5	10/07; 5/08
4	***B65***	**28**	**8**	9	2	10/05
2	***B67***	**28**	**8**	**11**	2	5/05; 10/05
2	***B57***	**28**	5	18	1	5/05
4	***B68***	**28**	**8**	28	2	10/08
2	***B72***	**28**	10	30	1	10/05
7	***R40***	**22**	**8**	**11**	2	5/07; 5/08
4	***R42***	**22**	**8**	**23**	2	10/08; 5/09
4	***R51***	**28**	**15**	**14**	2	10/08

Most abundant (*copious*) segment genotypes are in bold and underlined. Shadowed blocks contain variants that might have derived from 1–2 point mutations in one or two segments (e.g., strains *A13* and *A16* could have originated by accumulating one or two mutations, respectively, in the L segment of *A11* strains).

**Table 5:** Proportion of *copious* segment genotypes that composed the PUUV variants detected during the five-year study period.

Variants	Genogroup	no. of variants	% of *copious* genotypes of S	% of *copious* genotypes of M	% of *copious* genotypes of L
**The most frequently occurring (≥5 times)**	**A**	3	100	100	100
	**B**	6			
**Repeatedly observed (2–4 times)**	**A**	11	74.1	66.7	29.6
	**B**	16			
**Transient ( = 1 time)**	**A**	32	44.6	58.7	50
	**B**	60			
**Reassortant**		56	75	66.1	53.6

### Reassortant variants

Co-circulation of two distinct genogroups of PUUV allowed for straightforward recognition and subsequent study of reassortants variants. In total, 68 of 356 (19.1%) PUUV genomes were found to be reassortant. They were represented by 56 distinct variants and only three of those were observed more than once throughout the study period (Table 3). All six possible combinations of PUUV genome segments were found among reassortant strains and, interestingly, a certain pattern of segment combination was observed. In the vast majority of cases (95.6%) only the S or M segments were exchanged (Table 6). In other words, the L segment preferably remained paired with S- or M-segments of the same genogroup. Reassortant variants consisted of *copious* S- and M-segment genotypes in 75% and 66% of cases respectively, whereas the L genome segment was equally composed of *copious* and *sporadic* genotypes (Table 4). The composition of *copious* and *sporadic* genotypes was similar for both repeatedly observed and reassortant variants. However, the repeatedly observed variants could be seen at two different time-points, while reassortants were transient with the exception of three variants detailed in Table 5. The proportion of reassortants in the viral population varied from spring to autumn and was inversely related to PUUV prevalence, i.e. was higher in autumn when the prevalence is lower, and lower in spring when the prevalence is higher (Table 1). These oscillations were not observed for parental variants.

**Table 6:** PUUV Reassortants.

Combination of segments (S/M/L)	no. of genomes	Reassortant pattern	no. of genomes (% of total number of reassortants)
**ABA**	6	**S-L**/M	33 (48.5)
**BAB**	27		
**ABB**	10	**M-L**/S	32 (47.1)
**BAA**	22		
**AAB**	2	S-M/**L**	3 (4.4)
**BBA**	1		

Pattern for combination of genome segment. S, M and L stand for Small, Medium and Large genome segments, respectively.

### Double infections

A prerequisite for PUUV reassortment is the coexistence of two viral genomes in the same host, i.e. a double infection. Visual inspection of sequence chromatograms revealed double peaks at the diagnostic positions for genogroups in twelve sequences: six for the S segment, two for the M segment and four for the L segment. Double peaks were distributed not randomly along the sequences but appeared exclusively at the genotype-specific positions. Hence, such double peaks cannot result from simultaneous nt substitution at given positions reflect a double-infection. Detected double infections with two genotypes were not observed circulating as reassortant variants. Sequencing of individual cDNA clones showed the presence of two strains of the virus (one of each genogroup) and hence confirmed a double infection in the host. Interestingly, all but one sequence with double peaks were recovered from samples collected in October when conditions for double infections seem to be higher (see Discussion).

To search for traces of recombination in PUUV genomes circulating at Konnevesi, four variants, two of each genogroup (A and B), were selected and their complete S and M segment sequences and approx. 80% of the L segment sequence were recovered and analysed. No apparent signs of recombination were observed. Although this result does not completely exclude the possibility of recombination, it suggests that recombination occurs much less frequently than reassortment. An alternative explanation could be that recombinant viruses, even if generated frequently, may not be able to replicate as efficiently as parental viruses or reassortants.

## Discussion

For the first time, the microevolution of PUUV has been studied throughout the host population cycle. The data set analyzed here (1369 bank voles of which 360 were PUUV infected) was indeed larger than in our preceding study [19], in which 147 bank voles captured in 2005 and 40 PUUV genomes were analyzed. The present results support and expand our earlier conclusions concerning PUUV genetic diversity and the frequency of segment reassortment. In addition, the five-year monitoring of PUUV genomes allowed the study of PUUV microevolution, particularly, to follow the fate of individual genetic variants, including reassortants.

Of 1369 bank voles captured, 360 were found infected and 356 PUUV genome sequences were successfully recovered. Although not statistically significant, PUUV prevalence tend to be higher in the spring when most bank voles were mature and had over-wintered, than in the autumn when animals born the summer before predominate (Table 1). This observation is in concordance with other studies on PUUV prevalence [18], [36]–[37].

Earlier analyses of circulating PUUV in the same host population at Konnevesi revealed two distinct genogroups, A and B [19]. Both genogroups were present in the viral population along the monitoring period with a sole exception; no variants of the group B were detected in October 2009 (Table 1 and Fig. 3). It cannot be clarified here if this is a reflection of the low numbers of that sample set or that genogroup suddenly disappear. Genogroup B was generally more abundant and diverse (Table 1 and Fig. 3). The ratio of circulating A and B variants was relatively stable through the host density cycle, except during the decline phases in 2006 and 2009 (Table 1), and no clear signs of competition between genogroups were detected during the study. To our knowledge, competition between hantavirus variants has only been studied in cell culture [38]–[39] and corresponding data for other RNA viruses are limited (e.g. [40]).

The PUUV population was composed of several segment genotypes, L genotypes being more numerous than S or M genotypes. The range of genetic diversity within each genogroup was comparable for all three segments and, for the S and M segments also the inter-genogroup diversity was similar. In contrast, L-segment sequences showed surprisingly high diversity between the two genogroups (Table 2). It should be emphasized that the variability of the L segment region selected for our analysis (nt 505–1040) is typical of the segment among PUUV strains. Therefore, a likely explanation for the high inter-genogroup diversity of L segment could involve an independent evolutionary history of those genome segments of the Konnevesi strains. Phylogenetic analysis supported this hypothesis: the L sequences of genogroup A shared a MRCA with PUUV Sotkamo strain while genogroup B shared the MCRA with PUUV Pallasjärvi strain, both strains (i.e., certain genetic variant of a virus species) belong to the Finnish genetic lineage (Fig. 1A–C). Different evolutionary histories for PUUV genome segments were earlier suggested to account for observed variation in the Alpe-Adrian [41] and Latvian [42] lineages. Incidentally, 60 km south-east of the Konnevesi study area, at Pieksämäki, only variants of genogroup A were observed [43]. Whether this is a consequence of competition between A and B variants or peculiarity of their geographical distribution remains unclear. Extended monitoring of the PUUV variants in circulation is required to clarify this issue.

In Finland, PUUV prevalence fluctuates seasonally. However, this study shows that genetic composition of the viral population is not dependent of such seasonality. At each sampling point the PUUV population was roughly renewed; some genetic variants (i.e., sets of three genome segments) were observed persisting throughout the study but a large number were observed only temporarily (Table 3). In contrast, many of the segment genotypes were detected throughout the entire study period (Fig. 3). In other words, the survival of segment genotypes seems independent of the circulating variants, suggesting that genome segments persists independently and not in combination with other segments within the viral population. A factor that could account such outcome may be the frequent reassortment between PUUV variants, both closely- and distantly-related.

The most frequently occurring PUUV variants were always composed of *copious* genotypes. Transient variants seem to result from *copious* segment genotypes by accumulating nt substitutions, suggesting that viral genome segments are continuously drifting to provide the PUUV population with a diverse base from which to respond to new stressors. The three reassortant variants that were repeatedly detected throughout the study were composed of *copious* segment genotypes (Table 4), suggesting that they have a higher chance of co-infecting a host and/or have a pronounced ability to replace *sporadic* segment genotypes in a double infection scenario. Variants composed of *sporadic* segment genotypes are likely to be replaced when a co-infection occurred, thus only the dominant (i.e., *copious*) variants remain.

A substantial portion of co-circulating PUUV strains (19.1%) possessed a reassortant genome. Altogether, 56 distinct reassortant variants were observed. The majority of reassortants (53 of 56) were transient, suggesting that reassortants did not outcompete parental variants. The analysis of a large number of PUUV genomes allowed for the detection of all six possible reassortant types (Table 6). This finding proved that all reassortant types are viable in natural conditions, agreeing with the *in vitro* reassortment of orthobunyaviruses [44]. Different PUUV reassortants were found with different frequencies: in the vast majority of reassortants (95.6%), either L and S segments or L and M segments belonged to the same genogroup. Such a pattern of segments' combination suggests that reassortant types may differ in their replication efficacy. Unique reassortant variants were observed during every sampling event of the study (except 2006, when no PUUV was detected), and tended to be more prevalent in the autumn, even-tough the rates differences were not statistically significant. Seasonality of reassortment could be explained by seasonal variations in the age structure of the host population. During autumn, most bank voles are younger and recent infections are more likely to occur during a narrow time-window. Hence, double infections in naive bank voles become more likely and the conditions more favorable for reassortment. Indeed, 11 of the 12 double infections were detected in the autumn samples, whether were shed as parental or reassortant strains remain unknown.

Apparently, only inter-species, inter-lineage or inter-genogroup PUUV reassortants could be clearly recognized. In nature, when genome segments are exchanged between closely related variants (i.e., between variant of the same genogroup), reassortants might be very difficult to detect. Such “imperceptible” reassortment could be a useful mechanism to maintain a steady state in the PUUV population. For example, it could counteract the effects of ‘Muller's ratchet’ [45]–[46]. Experiments with the segmented RNA Φ6 virus support the notion that reassortment can reduce an excessive mutational load in a population and hence helps to avoid the accumulation of deleterious effects [47]–[48].

To conclude, for the first time the microevolution of a hantavirus was studied throughout a population cycle of its host. Analyses of 356 PUUV genomes circulating in a bank vole population over a five-year period allowed to study the mechanisms of viral genetic diversification and follow the fate of variants. The few PUUV variants that survived over several seasons did not show any sign of a founder effect. Collectively, the observations supported a quasi-neutral mode of PUUV microevolution with a steady generation of transient variants, including reassortants, and preservation of a few preferred genetic variants over several seasons/years.
